# The Viral Macrodomain Counters Host Antiviral ADP-Ribosylation

**DOI:** 10.3390/v12040384

**Published:** 2020-03-31

**Authors:** Yousef M. O. Alhammad, Anthony R. Fehr

**Affiliations:** Department of Molecular Biosciences, University of Kansas, Lawrence, KS 66045, USA; alhammad.yousef@ku.edu

**Keywords:** ADP-ribose, macrodomain, PARPs, stress granule, ADP-ribosylation, RNA virus, alphaviruses, coronaviruses, hepatitis E virus

## Abstract

Macrodomains, enzymes that remove ADP-ribose from proteins, are encoded by several families of RNA viruses and have recently been shown to counter innate immune responses to virus infection. ADP-ribose is covalently attached to target proteins by poly-ADP-ribose polymerases (PARPs), using nicotinamide adenine dinucleotide (NAD+) as a substrate. This modification can have a wide variety of effects on proteins including alteration of enzyme activity, protein–protein interactions, and protein stability. Several PARPs are induced by interferon (IFN) and are known to have antiviral properties, implicating ADP-ribosylation in the host defense response and suggesting that viral macrodomains may counter this response. Recent studies have demonstrated that viral macrodomains do counter the innate immune response by interfering with PARP-mediated antiviral defenses, stress granule formation, and pro-inflammatory cytokine production. Here, we will describe the known functions of the viral macrodomains and review recent literature demonstrating their roles in countering PARP-mediated antiviral responses.

## 1. Identification of Viral Macrodomains

**Figure 1:**
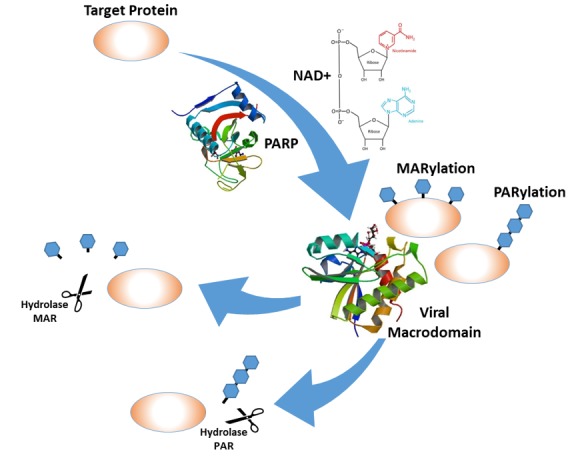
A schematic representation of the ADP-ribosylation and de-ADP-ribosylation by the viral macrodomains: The crystal structures of the poly-ADP-ribose polymerase (PARP)-12 protein [10] were downloaded from the protein data bank (PDB) (doi:10.2210/pdb2PQF/pdb), and the Venezuelan equine encephalitis virus (VEEV) macrodomain structure [11] was downloaded from the PDB (doi:10.2210/pdb5mqx/pdb).

Viral macrodomains are small protein domains of about 15–20 kDa encoded within the nonstructural proteins of several RNA viruses. Computer-assisted comparisons of RNA viruses in the early 1990s identified a conserved region of known function in the polyproteins of the Coronaviridae, Togaviridae, Matonaviridae, and Hepeviridae families which was named the “X” domain [1,2]. Eventually, the “X” domain was renamed macrodomain based on the protein folding that appear to be similar to the “macro” part of the macroH2A protein. The macrodomain is encoded within nonstructural protein 3 (nsP3) of the coronaviruses and alphaviruses and within open reading frame 1 (ORF1) of the rubella virus and hepatitis E virus. Several crystal structures of alphavirus and coronavirus macrodomains have been determined and demonstrate a highly conserved α/β/α sandwich fold [3,4]. The biochemical function of viral macrodomains were ambiguous until the discovery that viral macrodomains are enzymatically active and bind to poly- and mono-ADP-ribose [4,5,6,7]. Viral macrodomains were originally shown to have ADP-ribose-1”-phosphatase activity, removing phosphate from ADP-ribose-1”-phosphate. However, more recently, it has been demonstrated that they have hydrolase activity that removes ADP-ribose from proteins (Figure 1) [8,9].

## 2. ADP-Ribosylation and the Innate Immune Response

ADP-ribosylation is a posttranslational modification where ADP-ribose molecules are covalently attached to target proteins at one of several different amino acids including glutamate, aspartate, cysteine, lysine, arginine, and serine [12,13]. Additionally, it has been shown that ADP-ribose molecules can be added to nucleic acids [14]. The ADP-ribose is transferred from nicotinamide adenine dinucleotide (NAD+) onto target proteins as a single molecule of ADP-ribose (mono-ADP-ribose (MAR)) [8] or as consecutive individual units to form polymers of ADP-ribose molecules (poly-ADP-ribose (PAR)) by ADP-ribosyl transferases (ARTs) including the poly-ADP-ribose polymerases (PARPs) [15]. There are 17 known PARPs in the human genome, and more than half of them are induced by interferon (IFN), implicating ADP-ribose in the antiviral defense system. When IFN binds to its receptor, the IFN α/β receptor (IFNAR), it initiates a signaling cascade that results in the transcription of hundreds of interferon-stimulated genes (ISGs), many of which have antiviral activities. PARPs have many well-known pro- and antiviral activities (reviewed in Reference [16]). For instance, PARP12 is required for the ADP-ribosylation of Zika virus proteins NS1 and NS3 that inhibit Zika virus replication [17]. Conversely, PARP7 ADP-ribosylates TBK-1 which inhibits IFN production and leads to enhanced replication of influenza virus [18].

## 3. ADP-Ribose Binding and Hydrolase Activities of the Viral Macrodomains

**Figure 2:**
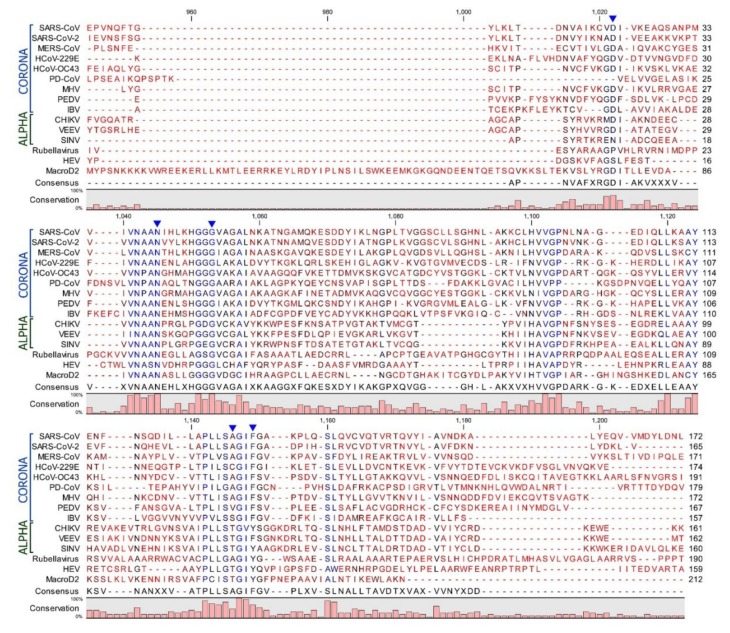
Sequence alignment of the amino acid sequences of various viral macrodomains from coronaviruses, alphaviruses, rubellavirus, and hepatitis E virus: The human macroD2 protein sequence was included for comparison. Sequences of viral and human macrodomains were alignment using CLC Genomics Workbench software. Arrows indicate residues that are discussed in the text.

ADP-ribosylation is a reversible modification via several enzymes that belong either to the ADP-ribosylglycohydrolase (DraG-like) family or to the macrodomain family [19]. Some of these enzymes hydrolyze a single unit of MAR, whereas the poly-ADP-ribose glycohydrolases (PARGs) can remove polymers of ADP-ribose molecules at *O*-glycosidic bonds [20,21,22,23]. It has been described that these enzymes hydrolyze ADP-ribose from target proteins at specific amino acid positions [24]. Human macroD2 enzymes, for instance, remove ADP-ribose from MARylated proteins at glutamate-ADP-ribose linkages [20,21]. Sequence analysis of viral macrodomains place them in the macroD2 family (Figure 2) and suggests that de-MARylation may be the primary enzymatic activity of the viral macrodomains [8].

Early structural data demonstrated that macrodomains, including viral macrodomains, are ADP-ribose-binding proteins [3,4,7]. They bind to both MAR and PAR, though the Hepatitis E virus (HEV) macrodomain required the inclusion of the downstream helicase domain of ORF1 to bind PAR [8]. Comparisons of ADP-ribose binding between the cellular and viral macrodomains were described in several studies [3,4,7] and, thus, will not be extensively discussed here. However, we will discuss some of the basic principles of ADP-ribose binding and hydrolysis by viral macrodomains.

First, as stated above, all viral macrodomains can bind to both MAR and PAR. These enzymes are efficient erasers of MAR but are less efficient at removing PAR [8,9]. It is yet unclear which of these activities play a dominant role during virus infections, but current data suggests that de-MARylation is their primary function. Data from McPherson et al., Abraham et al., and Fehr et al. support this idea as these studies demonstrated a correlation between de-MARylating activity and virus replication in cell culture and pathogenesis in vivo [25,26,27]. Also, the IFN-inducible PARPs are mono-ADP-ribosylating PARPs, again suggesting that viral macrodomains likely act to counter this antiviral response. However, it is also conceivable that MAR and PAR may work together in the antiviral response, as PARylation of Zika virus proteins has been observed, which is dependent on PARP12 activity. However, it is not clear how the MARylating activity of PARP12 is connected to the PARylation of Zika virus proteins [17].

Mutagenesis studies have identified several conserved residues within the macrodomain that impact ADP-ribose binding and/or enzymatic activity (summarized in a previous review [28]). Most notably, the distal ribose is tightly coordinated by two distinct loop regions that are highly conserved. Loop 1 includes a highly conserved triple glycine motif that interacts with the α-phosphate and the 1” and 2” OH groups of the distal ribose. Loop 2 contains a highly conserved GIF (Coronaviruses (CoVs)) or GIY (Togaviruses) motif that primarily contacts the β-phosphate of ADP-ribose and provides van der Waals contacts to direct the orientation of the distal ribose. A recent NMR structure, which depicted ADP-ribose bound and unbound forms of the VEEV macrodomain, showed that these loops undergo a dramatic conformational shift upon encountering ADP-ribose [11]. This is especially true for loop 1, which moved close to 3Å during this transition. A highly conserved asparagine residue (N41 in severe acute respiratory syndrome (SARS)-CoV) also appears to help coordinate the distal ribose, while an aspartic acid (D23 in SARS-CoV) provides hydrogen bonds to contact the amino group of the adenine. However, this aspartic acid is not completely conserved, as a serine or asparagine can provide these contacts in some alphaviruses (Figure 2).

**Figure 3:**
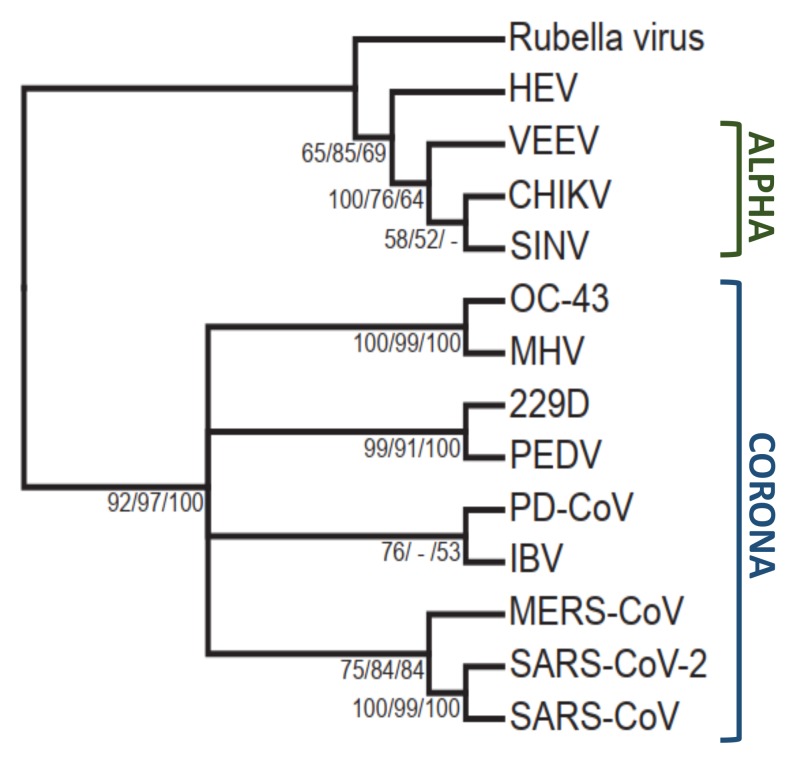
Phylogenetic tree of the viral macrodomains proteins sequences constructed with the neighbor joining method using Geneious Bioinformatics software.

The mechanism of de-ADP-ribosylation is not fully understood but likely involves the proper coordination of the protein-ADP-ribose bond near a conserved catalytic water molecule that can complete hydrolysis of the bond. The chikungunya virus (CHIKV) macrodomain was only able to hydrolyze ADP-ribose from acidic residues, indicating that viral macrodomains may only be able to cleave ester bonds. A mutation in loop 2 has been identified in both CHIKV and Sindbis virus (SINV), Y114A, that has reduced hydrolysis but increased ADP-ribose binding activity, indicating that this residue may be specifically important for hydrolysis. These mechanisms may not be identical between CoVs and alphaviruses, as they are phylogenetically distinct (Figure 3). Additional structural and mutagenesis studies will be needed to determine the precise mechanism of hydrolysis for macrodomain proteins.

## 4. The Role of Macrodomains in Virus Replication In Vitro and In Vivo

The impact of viral macrodomains in viral replication and pathogenesis has been extensively examined over recent years, utilizing reverse genetic systems for multiple alphaviruses, coronaviruses, and HEV. Here, we will briefly discuss the results from each viral family that has been tested.

### 4.1. Hepatitis E Virus

HEV is the sole member of the Hepeviridae family and is a non-enveloped positive-sense RNA virus with 3 ORFs. It is an emerging pathogen and possibly the most common cause of acute viral hepatitis in the world, with up to 3 million infections per year. HEV infection can lead to chronic hepatitis and cirrhosis and can cause stillbirths of the fetus in pregnant women [29]. The ORF1 polyprotein of HEV contains multiple domains, including the macrodomain, which is adjacent to RNA helicase domain. Interestingly, the RNA helicase domain was shown to be important for the ability of the macrodomain to bind and hydrolyze PAR and MAR [8]. HEV replicates poorly in cell culture, so reverse genetic systems for this virus have largely utilized replicon systems where HEV replicon plasmids containing a reporter construct are transfected into Huh-7 cells. Parvez tested the role of the HEV macrodomain during infection using a replicon with a GFP reporter and found that mutations in loop 1 and loop 2 and in the highly conserved asparagine residue eliminated GFP expression, while Li et al., using a luciferase reporter, also found that similar mutations in these same regions reduced luciferase activity [8,30]. These studies indicate that the HEV macrodomain is likely important for virus replication, though it would be of interest to determine its impact using virus replication systems for HEV, which are limited at this time.

### 4.2. Alphaviruses

Alphaviruses are enveloped, positive-sense RNA viruses and are prominent arboviruses that replicate in both vertebrate and invertebrate hosts. This family of viruses include human pathogens such as CHIKV, Ross River Virus (RRV), and Venezuelan equine encephalitis virus (VEEV). These viruses can cause a number of different pathologies, with severe arthritis or rash being the most common.

**Figure 4:**
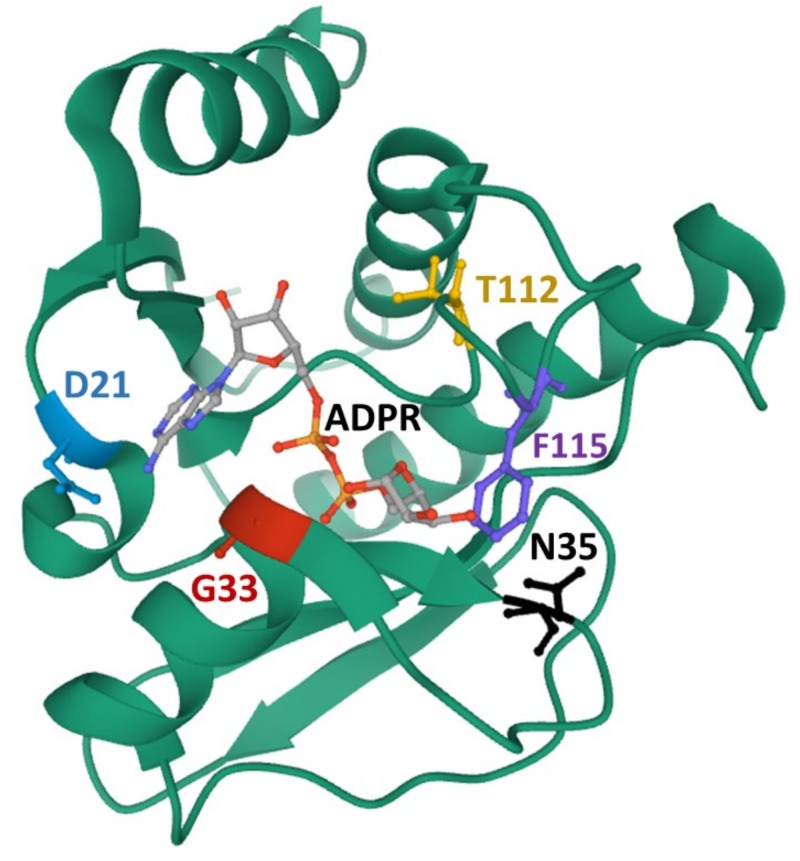
The crystal structure of the VEEV macrodomain protein [11] was downloaded from the PDB (doi:10.2210/pdb5mqx/pdb): Highlighted residues are D21 (blue, D20 in chikungunya virus (CHIKV)); N35 (black, N34 in CHIKV); G33 (red, G32 in CHIKV); T112 (yellow, T111 in CHIKV); and F115 (purple, Y114 in CHIKV). ADP-ribose (ADPR) is also shown.

The first studies on the role of the alphavirus macrodomain were in SINV, where viruses with mutations in highly conserved asparagine residues replicated normally in baby hamster kidney 21 (clone-13 cells) (BHK-21 cells) but were highly attenuated in vivo [31]. A more recent study using additional mutations in the catalytic hydrolase loop (N24 and G32) of the SINV macrodomain (Figure 4) came to similar conclusions while also showing that mutations that have reduced hydrolase and that binding activities replicate poorly or quickly revert in mouse neuronal cells. In addition, a Y114A mutation had slightly greater innate responses in the nervous system [32].

In slight contrast, CHIKV macrodomain mutations have severe effects on virus replication in all cell types [25]. Several recombinant viruses containing mutations that significantly affect hydrolase and binding activities were unrecoverable in BHK cells, as they quickly reverted back to wild-type sequence. A loop 1 mutant (G32E) even reverted following transfection into a mosquito cell line, indicating that antiviral ADP-ribosylation occurs in mosquito cells as well. To study the impact of the macrodomain in CHIKV infection, the authors made more subtle mutations in the macrodomain that allowed for the recovery of mutant viruses (G32S, G32A, T111A, and Y114A). While these mutants were only partially defective in hydrolase or ADP-ribose binding, they were severely attenuated in cell culture and in mice, demonstrating the incredible ability of ADP-ribosylation to restrict the replication of alphaviruses. Importantly, the Y114A mutation in SINV and CHIKV had reduced hydrolase activity but enhanced ADP-ribose-binding abilities. While attenuated compared to wild-type virus, this mutant was significantly more virulent than other mutations with similar hydrolase activity, indicating distinct roles for ADP-ribose binding and hydrolysis in pathogenesis.

G32S and Y114A were further utilized to identify the impact of ADP-ribose binding and hydrolase activities in the viral lifecycle [26]. The Y114A mutant virus, which only lacks hydrolase activity, initiated infection normally and produced similar levels of viral RNA and proteins compared to wild-type virus. However, these processes were dramatically reduced following infection of the G32S mutant virus, which is also defective in binding. This indicates that ADP-ribose binding but not hydrolysis activity is critical for early stages of the viral lifecycle. The lack of a defect in viral protein production but a significant drop in viral titers following infection with Y114A indicates that hydrolase activity may have a more substantial impact on later stages of the virus replication cycle.

More recently, it was shown that mutations V33E and D10A of the CHIKV macrodomain, which likely impair both ADP-ribose binding and hydrolysis [9,25], affected polyprotein processing by the nsP2 protease. In vitro, it was shown that the protease could be ADP-ribosylated by PARP10, that protease activity was altered by ADP-ribosylation, and that the macrodomain could reverse this modification [33]. It will be of great interest to determine if nsP2 is ADP-ribosylated during infection.

### 4.3. Coronaviruses

Coronaviruses (CoVs) are large, enveloped, positive-sense RNA viruses that cause a variety of diseases in humans and veterinary animals [34]. Human CoVs, such as hCoV-229E, cause ~10%–30% of common cold cases but, until the early 2000s, were not thought to cause serious diseases in humans. However, in the past 2 decades, 3 new pathogenic CoVs have emerged in China and the Middle East that cause deadly human infections. These include the severe acute respiratory syndrome (SARS)-CoV, Middle East respiratory syndrome (MERS)-CoV, and the ongoing pandemic outbreak of SARS-CoV-2. The coronaviruses macrodomain is located within nsp3, and its role in replication or pathogenesis has been analyzed in hCoV-229E, murine hepatitis virus (MHV), SARS-CoV, and infectious bronchitis virus (IBV). Most studies of the CoV macrodomain utilize mutation of the highly conserved asparagine residue (N41-SARS-CoV) to alanine, which has been shown to ablate both the phosphatase and hydrolase activity of the macrodomain [4,5,27]. Normal virus replication in cell culture was observed in almost all cases, including hCoV-229E infection of human fetal lung fibroblast cells (MRC-5 cells) [5]; IBV infection of chicken kidney and chicken embryonic fibroblasts [35]; MHV-A59 infection of peritoneal macrophages, Kupffer cells, dendritic cells, and L929 cells [36]; MHV-A59 and MHV-JHM (two distinct strains of MHV) infection of 17CL-1 cells [36,37]; and SARS-CoV infection of Vero and Calu-3 cells (a bronchial epithelial cell line) [27,38]. In addition, the complete deletion of the SARS-CoV macrodomain did not significantly affect luciferase expression in a SARS-CoV replicon [39]. These results indicate that the hydrolase activity of the macrodomain is not a general requirement for CoV replication.

However, the MHV-JHM asparagine-to-alanine mutation (N34A) replicated poorly in bone-marrow-derived macrophages (BMDMs) [40]. BMDMs are primary cells that elicit a robust innate immune response, indicating that the macrodomain may function to counter the innate antiviral immunity. Consistent with this idea, the MHV-JHM, SARS-CoV, and hCoV-229E mutant viruses were sensitive to interferon (IFN) pretreatment and the replication of the MHV-JHM mutant virus could be restored in IFN α/β receptor knockout (IFNAR^−/−^) cells, which are unable to respond to IFN treatment [38].

Coronavirus macrodomain mutant viruses are also highly attenuated in vivo. Both MHV and SARS-CoV mutants were found to cause minimal disease in mice [27,36,37], and these mutants also had significantly reduced titers in their target organs (liver or brain for MHV and lungs for SARS-CoV). To further implicate the innate immune response in the attenuation of these viruses, the MHV-JHM mutant virus caused severe disease upon either depletion of microglia in the brain or upon infection of mice lacking the ability to respond to interferon (IFNAR^−/−^) [40,41]. These results demonstrate that the hydrolase activity of the CoV macrodomain is largely dispensable for virus replication but is clearly required for pathogenesis and likely promotes virulence by countering the mammalian innate immune response.

Furthermore, the CoV macrodomain has been shown to also inhibit the production of IFN during certain infections. The SARS-CoV macrodomain mutant virus elicited a strong IFN and pro-inflammatory cytokine response in the lungs of infected mice during the early stages of infection and following infection of Calu-3 cells [27]. Using a coinfection model, it was shown that the increased cytokine response was partially responsible for the attenuation of this virus in vivo. Similarly, the MHV-JHM macrodomain mutant virus also led to a dramatic induction of IFN in BMDMs, while the MHV-A59 mutant virus also lead to increased production of IFN in plasmacytoid dendritic cells [36,40]. It remains unclear how hydrolase activity inhibits cytokine production.

Finally, a recent paper by Deng et al. showed that the MHV macrodomain and the PLP2 domain (protease and deubiquitinase (DUB)) can physically interact with each other [42]. Temperature-sensitive mutants were identified in the macrodomain that decreased the stability of PLP2 and nsP3 and negatively regulated replication and pathogenesis of MHV. Both domains regulate IFN production, and a direct interaction indicates that they may either regulate the same protein(s) that are dually regulated by ubiquitination and ADP-ribosylation or perhaps that the protease is ADP-ribosylated and this modification needs to be removed for its function.

## 5. PARPs Enhance IFN Production and Can Restrict Coronavirus Replication Following Macrodomain Mutant Virus Infection

While PARP enzymes were the likely mediators of antiviral ADP-ribosylation, other enzymes can ADP-ribosylate proteins. To determine that PARPs were indeed inhibiting MHV infection in the absence of the macrodomain, wild-type (WT) and mutant virus-infected BMDMs were treated with PARP inhibitors (3-AB and XAV-939), both of which can inhibit multiple PARPs, including both PARylating and MARylating PARPs [43]. These inhibitors substantially increased the replication of the macrodomain mutant virus without having any noticeable effect on the WT virus. Additionally, the PARP inhibitors decreased the level of IFN-β production back to WT levels, further demonstrating that PARP-mediated ADP-ribosylation plays a role in regulating IFN production during infection [40].

It is well known that PARPs are upregulated by IFN [44]. Recently, the expression of all 17 mammalian PARPs in BMDMs following MHV infection was examined to identify PARPs that could potentially be involved in restricting its replication. Using quantitative RT-PCR, 7 of the 16 mouse PARPs were highly expressed and dramatically upregulated following MHV infection. Their expression was mostly absent in IFNAR^−/−^ cells, consistent with their strong upregulation by IFN. To identify the specific PARP(s) that inhibits virus replication, an siRNA screen was then performed. Knockdown of both PARP12 and PARP14 partially restored mutant virus replication while having no impact on wild-type virus. All other PARPs tested were unable to restore mutant virus replication. In addition, PARP14 promoted IFN production during CoV infection or poly(I:C) treatment, consistent with another study showing that PARP14 enhanced IFN production following treatment of macrophages with lipopolysacharacide (LPS) stimulus [45]. These data are consistent with the hypothesis that PARP12 and PARP14 are coronavirus restriction factors, but their functions are largely mitigated by the viral macrodomain.

## 6. The Alphavirus Macrodomain Can Block Stress Granule Formation

Stress granules (SGs) are non-membranous structures containing a mixture of RNA and protein including stalled translation initiation complexes that form upon cellular stress. One of these types of stress is viral infection. Early stages of many virus infections result in the formation of dsRNA that can activate protein kinase R (PKR). PKR then phosphorylates eIF2α, blocking mRNA translation, which then promotes the assembly of SGs [46]. These stress granules limit the amount of translation factors available, which potently interferes with virus replication as all viruses require cellular protein machinery to translate their proteins [47]. SGs are regulated by multiple different types of posttranslational modifications, including ADP-ribosylation. As such, SGs contain several PAR glycohydrolase isoforms, along with ADP-ribosylated proteins such as the RNA decay factor protein, G3BP1. In addition, several PARPs has been identified within the stress granules (PARP-5a, PARP-12, PARP-13, PARP14, and PARP-15); however, SG proteomes rarely identify all 5 of these PARPs, and so PARP composition dynamics within SGs likely differ according to multiple factors including cell type and type of stress [48,49,50].

Stress granule formation and disassembly are highly regulated during viral infection. Viruses use different strategies to disrupt SGs, either by suppressing their formation or by promoting their disassembly. Ebola virus, for instance, inhibits SG initiation through interactions of the C-terminal domain of the VP35 protein with different components of the SGs including G3BP1, eIF3, and eEF2 [51]. However, in many cases, the mechanism of such action is unknown.

Alphaviruses, such as CHIKV, induce stress granule formation at the early stage of the viral infection followed by the disassembly of SGs which is mainly influenced by nsP3 [52]. nsP3 contains the macrodomain, a zinc-binding domain, and a C-terminal hypervariable region domain (HVD). The hypervariable domain (HVD) was previously known to interact with G3BP1 and G3BP2, and thus, it was proposed that this interaction results in sequestration of G3BP1/2 and SG disassembly [53,54,55]. However, a recent study reported that the HVD of CHIKV alone was unable to affect SG formation, though it did interact with SGs. They did show that the full-length nsP3 was able to prevent the accumulation of SGs, suggesting that another factor in nsP3 besides the HVD is involved in SG disassembly. Interestingly, overexpression of the CHIKV macrodomain alone inhibited SG formation induced by multiple cell stressors. This study further showed that hydrolase activity of the macrodomain was required for the ability to disrupt SGs. Additionally, hydrolase activity was required to prevent arsenite-induced SG formation during a CHIKV infection. Finally, the authors hypothesized that the macrodomain could remove ADP-ribose from G3BP1, a critical factor for SG formation that is known to be ADP-ribosylated. Upon overexpression of G3BP1 in 293F cells, ADP-ribosylation of G3BP1 could be observed; however, this modification was lost upon overexpression of nsP3, suggesting that the macrodomain can remove ADP-ribose from G3BP1 [56]. Interestingly, there was a clear distinction in the composition of SGs that were disrupted by the macrodomain. The disrupted SGs still maintained mRNA-binding proteins but lost all proteins involved in translation, indicating that the macrodomain disrupts SGs in a specific manner. In conclusion, the results from this study show that SG formation is dependent on the ADP-ribosylation of proteins within SGs and that the hydrolase activity by the alphaviruses macrodomain is able to disassemble SGs.

## 7. Future Directions for Macrodomain Research

Studies from the past 15 years have clearly shown that viral macrodomains bind MAR- and PARylated proteins and enzymatically hydrolyze ADP-ribose from proteins. More recently, it has been demonstrated that macrodomains likely counter PARP activity, block IFN production, and can disassemble stress granules. However, the biological processes and target proteins of viral macrodomains remain largely unknown. Based on the evidence described here, it is clear that the de-ADP-ribosylating activity of the macrodomain is critical for its functions. However, it cannot be ruled out that some of these mutations discussed herein may impact other macrodomain functions, such as nucleic acid binding, which could also impact virus replication.

While the CHIKV macrodomain can de-ADP-ribosylate G3BP1 and the nsP2 protease when each is overexpressed in mammalian cells or in vitro, it is unclear if these proteins are targeted by viral macrodomains during infection. Macrodomains surely target multiple proteins due to the number of different phenotypes associated with mutant viruses, so it will be of great interest to identify these proteins, to determine what cellular processes are affected by ADP-ribosylation, and to ultimately determine how ADP-ribosylation leads to inhibition of virus replication. However, finding these targeted proteins is challenging due to the number and diversity of ADP-ribosylated proteins in cells. Also, it should not be assumed that the functions of macrodomains from alphaviruses and CoVs or even within these virus families will be similar. Their protein sequences are significantly different (Figure 2 and Figure 3), and these viruses use distinct mechanisms for their replication and pathogenesis.

There is more work to be done to identify PARPs that are mediating antiviral ADP-ribosylation that is countered by macrodomains. PARP12 and PARP14 were identified as PARPs that could restrict replication of macrodomain mutant MHV in BMDMs, but it is likely that other PARPs may be important for different viruses or in different cell types. Identification of these PARPs may aid in the identification of meaningful ADP-ribosylated targets. Knowing what PARPs are mediating antiviral ADP-ribosylation may also lead to novel antiviral strategies.

It is also unclear whether the hydrolysis of MAR, PAR, or both is responsible for phenotypes associated with macrodomain mutant viruses. Despite a large number of macrodomain structures, the biochemistry of the macrodomain still requires further investigation to determine the mechanism of hydrolysis. It is also unclear whether macrodomains have specificity for certain proteins. Many in vitro assays for hydrolase activity utilize the de-ADP-ribosylation of auto-ADP-ribosylated PARP proteins, indicating that the macrodomain may be able to de-ADP-ribosylate most proteins it can interact with [8,9,25,27,32].

Finally, an intriguing question that remains is as follows: are macrodomains suitable targets for antiviral therapeutics? Considering the significant attenuation seen with macrodomain mutants, it seems that the answer is yes. However, humans also have macrodomains, so there is the potential for significant side effects. It will be important to identify biochemical differences between human and viral macrodomains, such that antiviral therapeutics could be developed that are highly specific for viral macrodomains. Alternatively, these therapeutics could be useful in an agricultural setting for highly virulent infections such as porcine epidemic diarrhea virus (PEDV)-infected pigs. As exemplified by the recent SARS-CoV-2 outbreak, developing therapeutics targeting highly conserved viral proteins, such as the macrodomain, could be a useful for creating an arsenal of drugs that could be deployed to treat emerging alphavirus, hepatitis E, or coronavirus infections.
